# On Biophysical Properties and Sensitivity to Gap Junction Blockers of Connexin 39 Hemichannels Expressed in HeLa Cells

**DOI:** 10.3389/fphys.2017.00038

**Published:** 2017-02-09

**Authors:** Anibal A. Vargas, Bruno A. Cisterna, Fujiko Saavedra-Leiva, Carolina Urrutia, Luis A. Cea, Alex H. Vielma, Sebastian E. Gutierrez-Maldonado, Alberto J. M. Martin, Claudia Pareja-Barrueto, Yerko Escalona, Oliver Schmachtenberg, Carlos F. Lagos, Tomas Perez-Acle, Juan C. Sáez

**Affiliations:** ^1^Departamento de Fisiología, Pontificia Universidad Católica de ChileSantiago, Chile; ^2^Centro Interdisciplinario de Neurociencia de Valparaíso, Universidad de ValparaísoValparaíso, Chile; ^3^Institute of Biomedical Sciences, Faculty of Medicine, Universidad de ChileSantiago, Chile; ^4^Computational Biology Lab (DLab), Fundación Ciencia & VidaSantiago, Chile; ^5^Department of Endocrinology, School of Medicine, Pontificia Universidad Católica de ChileSantiago, Chile; ^6^Facultad de Ciencia, Universidad San SebastiánSantiago, Chile

**Keywords:** Cx39, gap junction, electrical coupling, membrane potential, unitary conductance, dye-uptake, permeability

## Abstract

Although connexins (Cxs) are broadly expressed by cells of mammalian organisms, Cx39 has a very restricted pattern of expression and the biophysical properties of Cx39-based channels [hemichannels (HCs) and gap junction channels (GJCs)] remain largely unknown. Here, we used HeLa cells transfected with Cx39 (HeLa-Cx39 cells) in which intercellular electrical coupling was not detected, indicating the absence of GJCs. However, functional HCs were found on the surface of cells exposed to conditions known to increase the open probability of other Cx HCs (e.g., extracellular divalent cationic-free solution (DCFS), extracellular alkaline pH, mechanical stimulus and depolarization to positive membrane potentials). Cx39 HCs were blocked by some traditional Cx HC blockers, but not by others or a pannexin1 channel blocker. HeLa-Cx39 cells showed similar resting membrane potentials (RMPs) to those of parental cells, and exposure to DCFS reduced RMPs in Cx39 transfectants, but not in parental cells. Under these conditions, unitary events of ~75 pS were frequent in HeLa-Cx39 cells and absent in parental cells. Real-time cellular uptake experiments of dyes with different physicochemical features, as well as the application of a machine-learning approach revealed that Cx39 HCs are preferentially permeable to molecules characterized by six categories of descriptors, namely: (1) electronegativity, (2) ionization potential, (3) polarizability, (4) size and geometry, (5) topological flexibility and (6) valence. However, Cx39 HCs opened by mechanical stimulation or alkaline pH were impermeable to Ca^2+^. Molecular modeling of Cx39-based channels suggest that a constriction present at the intracellular portion of the para helix region co-localizes with an electronegative patch, imposing an energetic and steric barrier, which in the case of GJCs may hinder channel function. Results reported here demonstrate that Cx39 form HCs and add to our understanding of the functional roles of Cx39 HCs under physiological and pathological conditions in cells that express them.

## Introduction

Connexins (Cxs) are integral membrane proteins expressed in most tissues of vertebrate animals, and are coded in humans by 21 different genes (von Maltzahn et al., 2004). Six similar Cx subunits form a homomeric hemichannel (HC) and two similar Cx HCs form a homotypic gap junction channel (GJC), which enable communication between the intra and extracellular milieus or the cytoplasm of two adjacent cells, respectively (Sáez et al., 2003).

Cx HCs are involved in autocrine and paracrine cell signaling, because they are non-selective channels permeable to ions and different small molecules, including metabolic substrates and signaling molecules such glucose, NAD^+^, ATP, glutamate, NO and PGE_2_ (Bennett et al., 2003; Figueroa et al., 2013; Orellana and Stehberg, 2014). HC activity can be regulated by phosphorylation (Bao et al., 2007; Puebla et al., 2016), redox potential (Contreras et al., 2002; Retamal et al., 2006, 2007), pH (Beahm and Hall, 2002; D'hondt et al., 2013), and other conditions (Sáez et al., 2005). In addition, the open probability of different Cx HCs has been observed to increase in cells exposed to low extracellular Ca^2+^ concentration (Sáez et al., 2005), positive membrane voltages (Sáez et al., 2005) or mechanical stress (D'hondt et al., 2009), and is drastically reduced by La^3+^ and GJC blockers such as carbenoxolone (CBX), 18-β-glycyrrhetinic acid (BGA) and octanol among others (D'hondt et al., 2013). Nonetheless, it remains unknown whether the aforementioned conditions affect Cx39 HCs.

Cx HCs are believed to present low open probability under basal *in vitro* conditions (Trexler et al., 1996; Kondo et al., 2000; Contreras et al., 2003; Sáez et al., 2005). The range of unitary conductance of Cx HCs varies between 17 pS in Cx32 HCs (Gómez-Hernández et al., 2003; Oh et al., 2004) to 352 pS in Cx52 HCs (Valiunas and Weingart, 2000). The unitary conductance of Cx HCs is higher than that of Cx GJCs constituted of the same Cx type (Sáez et al., 2005). Specifically, the unitary conductance of homomeric HCs composed of Cx30, Cx43, or Cx45 is about twice the conductance of homotypic GJCs formed by the same Cx (Valiunas and Weingart, 2000; Valiunas, 2002; Contreras et al., 2003). Since the unitary conductance of Cx39 GJCs has not been described, it is not possible to speculate about the unitary conductance of Cx39 HCs.

The permeability of Cx HCs to molecules has been frequently studied by means of uptake or release of 1 to 3 small molecules including fluorescent dyes with different sizes, shapes and net charges (Schalper et al., 2008), such as ethidium bromide (Etd^+^), 4′,6-diamidino-2-phenylindole (DAPI^+2^) and Lucifer yellow (LY^−2^), among others (Sáez et al., 2005). With these tools, HCs composed of Cx26 (Figueroa et al., 2014), Cx32 (Sánchez et al., 2009), Cx43 (Contreras et al., 2003; Retamal et al., 2007; Orellana et al., 2011) as well as HCs formed by other Cxs described so far present similar permeability features to dyes upon activation with different stimuli. To date, Cx39 HCs express in HeLa cells have been shown to be permeable to Etd^+^ (Cea et al., 2013), but their permeability features remain largely unknown.

Mouse Cx39 shares ~61% sequence similarity with the human ortholog Cx40.1 and it is transiently expressed in mouse myoblasts during late embryonic development (von Maltzahn et al., 2004), after denervation of adult fast myofibers (Cea et al., 2013), as well as in *mdx* mouse myofibers (Cea et al., 2016), which is a model of Duchenne's disease. Cx39-deficient mice display accelerated myogenesis and regeneration of skeletal muscles (von Maltzahn et al., 2011). In exogenous expression systems, Cx39 does not form GJCs as determined by dye coupling techniques using a small number of permeability probes (von Maltzahn et al., 2004). However, formation of Cx39 GJCs has not been demonstrated with more sensitive techniques such as electrical coupling measurements.

In 2009, the structure of human Cx26 HCs was solved by X-ray crystallography, showing the formation of hexagonal transmembrane arrangements (Maeda et al., 2009). The HCs dock end-to-end to form an intercellular channel that spans the membrane bilayers of apposed cells and the virtual extracellular gap. Each monomer conformation is comprised of four transmembrane helices (TM1–TM4), in which any pair of adjacent helices are antiparallel, comprises two extracellular loops (E1, E2), one cytoplasmic loop (CL) and N-terminal helix (NTH) located intracellularly. This Cx structure can be studied with comparative modeling techniques (Koehler Leman et al., 2015), so as to gain additional insights into the structure-activity relationships of specific Cx isoforms and mutants involved in different pathologies (Harris, 2001; Araya-Secchi et al., 2012).

The present study includes functional assays for GJCs and HCs in HeLa cells transfected with Cx39 (HeLa-Cx39). We found that HeLa-Cx39 cells do not form functional GJCs since cells remain electrically uncoupled consistent with structural limitations identified by *in silico* studies, but present HCs activated by different conditions. These HCs are impermeable to Ca^2+^ but are permeable to small molecules with no clear preference for negative or positive net charges, and are inhibited by several conventional Cx-based channel blockers, but not others.

## Materials and methods

### Reagents

Octanol, heptanol, carbenoxolone, oleamide, LaCl_3_, Etd^+^, procion orange MX2R, brilliant blue G (BBG), Evans blue, propidium iodide, 4-Bromo calcium ionophore A23187 were purchased from Sigma-Aldrich (St. Louis, Missouri, USA). The 9-amino-6-Chloro-2-Methoxyacridine (ACMA), DAPI^+2^, hexidium iodide, nuclear yellow, BOBO 1-iodide, 7-aminoactinomycin D (7-ADD), YOYO 1-iodide, TOTO 1-iodide and ethidium homodimer–2–bromide obtained from Invitrogen (Massachusetts, USA), indocyanine green from Pulsion medical systems, (Feldkirchen, Germany) methylene blue from Euromed (Santiago, Chile).

### Cell lines

HeLa cells were cultured in a DMEM medium supplemented with 10% fetal bovine serum supplemented with 50 U/ml of penicillin and streptomycin, at pH 7.4 and kept at 37°C in 5% CO_2_/95% air atmosphere. HeLa-Cx39 and HeLa Cx43-EGFP transfected cells were selected with 0.5 mg/mL of puromycin and 0.3 mg of geneticin (Invitrogen, MA, USA), respectively, as described previously (Jordan et al., 1999; von Maltzahn et al., 2004). The Hela-Cx39 cells were kindly provided by Dr. Klaus Willecke, Lymes Institute, University of Bonn, Bonn, Germany. For each experiment, cells were seeded on glass coverslips, until appropriate confluence was reached. Cells were always fed the day before an experiment.

The Cx39 transfection was confirmed by immunofluorescence. In brief, cells on glass coverslips, were washed with PBS, and fixed with formaldehyde 4% and incubated with blocking solution (1% BSA, 0.025% triton X-100 and 50 mM NH_4_Cl, in PBS, at pH 7.4) overnight. Then, cells were incubated with rabbit anti-Cx39 antibody (Invitrogen, MA, USA) for 6 h at room temperature, washed with PBS and incubated 30 min with secondary rabbit anti-IgGs (Santa Cruz, Texas, USA) conjugated to Cy2 or Cy3. Preparations were subsequently washed with PBS, and coverslips were mounted with fluoromont G (Electron Microscopy Science, PA, USA).

### Electrophysiology

Functional HCs were evaluated in whole-cell voltage clamp experiments as previously described (Sánchez et al., 2009). The bath solution contained: (in mM) 140 NaCl, 5.4 CsCl, 1 MgCl_2_, 1.8 CaCl_2_, 2 BaCl_2_, and 10 HEPES at pH 7.4. The pipette solution contained (in mM): 130 CsCl, 10 AspNa, 0.26 CaCl_2_, 1 MgCl_2_, 2 EGTA, 7 TEA-Cl, and 5 HEPES at pH 7.2. The pipette resistance in the bath was 10–15 MΩ. All recordings were performed at room temperature (21–23°C). The microscopic and macroscopic currents were obtained with voltage steps (+60 mV for 40 s) and ramp protocols (−80 mV to +80 mV, for 9 s).

Membrane potential (RMP) measurements were performed under a whole-cell current clamp configuration at 37°C in individual cells. The pipette solution contained 3 M KCl and the bath solution contained: (in mM) 145 NaCl, 5 KCl, 3 CaCl_2_, 1 MgCl_2_, 5 glucose, 10 HEPES, 5 Tris at pH 7.4. The pipette resistance in the bath was 30–50 MΩ. The recorded RMP corresponded to the potential value measured when crossing the cell membrane and remained stable for 5 s after accessing.

For electrical coupling, high density cells cultures (50–70%) were bathed with a solution containing: (in mM) 150 NaCl, 4 KCl, 1 MgCl_2_, 1.2 CaCl_2_ and 5 HEPES at pH 7.4. The pipette solution was the same used in HC experiments plus 18 μM DAPI^+2^ in one pipette to identify the cell in indirect immunofluorescence for Cx39. The junctional current was recorded in an adjacent cell using voltage steps increasing in 20 mV from −100 to 0 mV and a duration of 200 ms each. The holding potential of non-stepped cells was −60 mV and the gap junction conductance (g_*j*_) was calculated from *g*_*j*_ = −*I*_*b*_/(*V*_*a*_ − *V*_*b*_), as described previously (Van Rijen et al., 1998). All experiments were done using an Olympus IX 51 inverted microscope with an Axopatch1-D amplifier, Digidata 1322 digitaizer and Clampex 9.1 acquisition software. Data were analyzed with Clampfit 2.1 software (Molecular Devices, CA, USA).

### Dye uptake assay

Cells were bathed in same control solution used for RMP experiments, supplemented with 5 μM of each dye that does not permeate the cell membrane (Table 1). Cells were recorded 5 min in control solution, and were then washed 5 times with control solution without CaCl_2_ and MgCl_2_ [divalent cation free solution (DCFS)] to promote HC opening, as shown for other Cx HCs (Cea et al., 2013). Images were recorded every 30 s for 20 min with a Nikon Eclipse Ti inverted microscope and NIS-Elements software (Nikon, Tokio, Japan); the analysis was done with ImageJ 1.42q software (NIH, MD, USA).

**Table 1:** **Dye uptake of in HeLa-Cx39 cells via HCs**.

**Commercial name**	**Molecular weight**	**Net charge**	**Dye uptake**	**Inhibited by HC blocker**
ACMA (9-Amino-6-Chloro-2-Methoxyacridine)	258.7	+1	–	n/a
Methylene blue	319.8	+1	+	+
DAPI^+2^	350.2	+2	+	+
Ethidium bromide	394.3	+1	+	+
Hexidium iodide	497.4	+1	+	+
Nuclear yellow	651.0	+1	–	n/a
Propidium iodide	668.3	+2	–	n/a
Procion Orange MX2R	715.5	−3	+	+
Indocyanine green	774.9	−1	+	+
BBG	854.0	−1	–	n/a
Ethidium homodimer	856.7	+4	–	n/a
Evans blue	960.8	−4	+	+
BOBO™-1 iodide	1,202.6	+4	–	n/a
7-AAD (7-Aminoactinomycin D)	1,270.4	0	+	+
YOYO®-1 iodide	1,270.7	+4	+	+
TOTO®-1 iodide	1,302.8	+4	+	+

*n/a, Not applicable*.

### Ca^2+^ signal

The intracellular free-calcium signal (hereinafter termed the Ca^2+^ signal), was recorded as previously described (Harcha et al., 2015). In brief, cells seeded on glass coverslips were loaded for 30 min at 37°C with 5 μM Fura-2AM (Invitrogen, MA, USA) in the same saline solution used for the dye uptake assay, and were then washed with the same solution without Fura-2AM. For Ca^2+^ signal measurements, fluorescence intensity was captured every 3 s. The fluorescence intensity ratio quantification (Ca^2+^ signal = F340/F380) and images were performed in a Nikon Eclipse Ti inverted microscope and imaged with NIS Elements software (Nikon, Tokio, Japan).

### Molecular modeling

Molecular models of both HCs and GJCs were produced using the human Cx26 (hCx26, PDB id: 2ZW3) crystal structure as a template (Maeda et al., 2009). Sequence alignments to produce molecular models were computed by introducing the amino acid sequence of the template, mouse Cx39 (mCx39) and mouse Cx43 (mCx43) in the phylogenetic analysis previously proposed by Araya-Secchi et al. (Abascal and Zardoya, 2013; Araya-Secchi et al., 2014). To do so, multiple sequence alignments were recomputed by Mafft v7 (Araya-Secchi et al., 2012) using the PAM200 substitution matrix, and the slow strategy with an all-pair local alignment. Sequence identity versus the template structure resulted in 31.3% and 42.3% for mCx39 and mCx43, respectively. Spatial constraints needed to produce the molecular models of mCx39 and mCx43 were compiled from the template structure by using MODELLER v 9.14 (Eswar et al., 2006). In total, 1000 models were produced for each target sequence, whose structural quality was assessed by using MAIDEN (Postic et al., 2015). MAIDEN evaluates adjustments of the interatomic distance between residues located at transmembrane helices in the lipid bilayer using its empiric energy function. The top scored model for each protein was selected for further analysis.

### Pore radius

Pore radius profiles for our molecular models and the template crystal structure were computed with the Epock cavity detector plugin (Laurent et al., 2015), as implemented in VMD (Humphrey et al., 1996). To do so, a prospective axis of 120 and 200Å in length for HCs and GJCs, respectively, was traced along the Z-axis of every molecular model. A scanning radius of 16Å was selected, where the available volume was computed by using a grid spacing of 0.5Å.

### Assessment of the electrostatic potential

The electrostatic potential of our molecular models was determined using PDB2PQR as implemented in the APBS 2.1 tool of PyMOL (Lerner and Carlson, 2006). This tool assigns hydrogen atoms and charges to molecular models according to the CHARMM forcefield. Then, the electrostatic potential was computed by using the Linearized Poisson-Boltzmann equation and default parameters. Figures were produced by using PyMOL's rendering capabilities.

### *In silico* dye modeling and characterization

All permeability probes were modeled using the Avogadro v1.0.3 suite (Hanwell et al., 2012). Molecular encoding was performed using the SMILES codes for each molecule obtained from NCBI's PubChem site (Bolton et al., 2008). When SMILES codes were not available, molecules were hand-drawn with Avogadro's drawing tool. All molecules were subjected to geometry optimization, performing an energy minimization procedure using the conjugate gradients algorithm and the MMFF94 forcefield (Halgren, 1996) within Avogadro. Next, a total of 1,875 molecular descriptors were calculated using the PaDEL-molecular descriptor program v2.21 (Yap, 2011). Descriptors that could not be computed for more than one molecule, or that were equal to 0 for all but one molecule, were discarded as non-informative. To select the descriptors that better represent the set of molecules, we used an approach based on K-nearest neighbors (KNN) classifiers combined with a genetic algorithm (GA) (Mitchell, 1996). Each GA-generated combination of descriptors was used to classify each molecule into two classes based on their ability to diffuse through mCx39 HCs. This classification was performed with a KNN classifier and a “leave-one-out” procedure. The GA employed works as follows: a population of 5,000 different subsets (i.e., genomes or solutions) of molecular descriptors was generated in the first iteration. Each of these genomes was a numerical vector of equal length to the total number of descriptors, in which the vector elements assigned to be used were set to 1, whereas those descriptors not used were set to 0. In the initial population, the first subset included all the descriptors that could be produced for all molecules (1,532 descriptors, all elements set to 1). The next subsets were formed by each descriptor, and the remaining subsets were randomly chosen as a combination of descriptors. Subsequently, the top 1,000 subsets that correctly classified a larger number of molecules, passed directly (and unaltered) to the next generation, and were used to create the remaining 4,000 solutions. These 4,000 solutions were created by random cross-over of the top 1,000 subsets, followed by point mutations (a switch of the value in the vector element to the other possible value) that occurred with a probability of 10^−3^. During the cross-overs procedures, a vector position was randomly chosen to combine two of the top 1,000 genomes, creating 2 child genomes. This protocol ensures fast convergence to subsets of descriptors that were able to correctly classify all the molecules using the KNN.

### Statistical analyses

All results are presented as mean ± SE and significant differences between groups were determined using a one-way ANOVA followed by a Tukey's multiple comparisons *post-hoc* test. Differences were considered significant at *P* < 0.05. Statistical analyses were performed using Excel 2013 (Microsoft, WA, USA) and GraphPad Prism 6 software (Graphpad software, CA, USA).

## Results and discussion

### HeLa-Cx39 cells do not form functional gap junction channels

**Figure 1:**
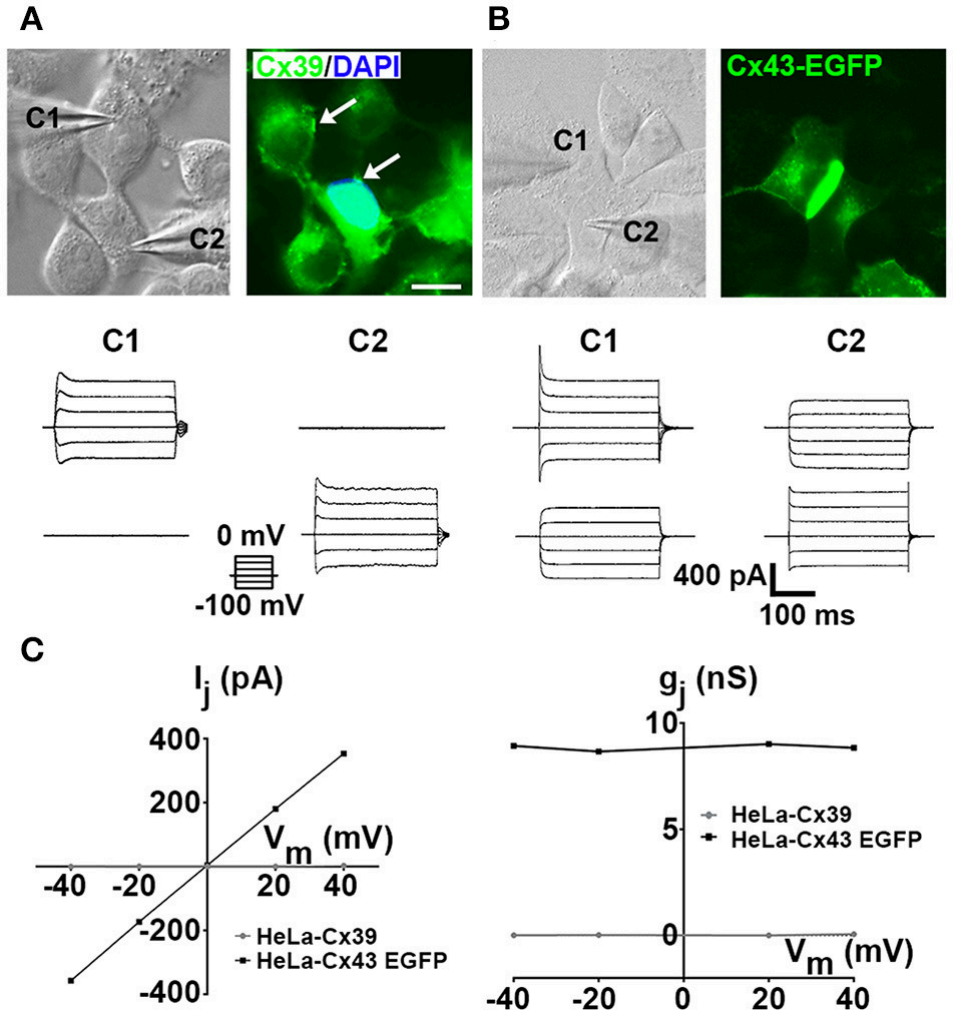
**HeLa-Cx39 cells do not form functional gap junctions. (A,B)** Phase contrast view (left) and immunofluorescence of Cx39 (**A**, right) and Cx43-EGFP fluorescence (Right) in HeLa-Cx39 and fluorescence of HeLa-Cx43EGFP cells (**B**, right). Bar scale: 20 μM. Arrows denote small Cx39 gap junctions plaques. **(C)** Junctional current (I_j_) at different voltages (left) and normalized junctional conductance (g_j_) at different voltages (right) using dual voltage-clamp measurements in HeLa-Cx39 and HeLa-Cx43EGFP cells. Values of I_j_ for HeLa-Cx39 cells were zero at all voltages studied. Records of intercellular current transfer from cell1 (C1) to cell2 (C2) and vice versa in HeLa-Cx39 cells (Below cells shown in **A**) and in HeLa-Cx43EGFP (Below cells shown in **B**) upon voltage steps (increasing in 20 mV from −100 to 0 mV with duration of 200 ms, left bottom traces) applied to C1 and C2, respectively (*n* = 9).

Cx43-EGFP fluorescence was intense at cell–cell contacts and more diffuse in perinuclear regions (Figure 1B right), consistent with functional cell–cell coupling (see below). In contrast, all HeLa-Cx39 cells showed Cx39 reactivity mainly as diffused intracellular labeling as well as small patches in the cell periphery and at apparent cell–cell interfaces (Figure 1A right, denoted by arrows), which does not rule out the possibility of functional gap junction formations. In addition, it remains to be confirm whether Cx39 from homotypic gap junctions using ultrastructure techniques, such immune-cryofracture or regular transmission electron microscopy. On the other hand, previous studies in HeLa-Cx39 cells have shown absence of dye transfer using Lucifer yellow, DAPI^+2^, propidium iodide, Etd^+^ or neurobiotin (von Maltzahn et al., 2004). However, it remained unknown if cells expressing Cx39 can establish electrical coupling, which is a much more sensitive way of detecting gap junctional communication.

The application of 20 mV voltage steps (from −100 to 0 mV with 200 ms duration) in HeLa-Cx43-EGFP cells generated evident transjunctional current (I_j_), which was not detected between HeLa-Cx39 cells (*n* = 9) (Figure 1C); values of I_j_ recorded in HeLa-Cx39 cells were zero at all voltages applied. Accordingly, and as previously described (von Maltzahn et al., 2004), intercellular DAPI^+2^ transfer was negative (Figure 1A right). Calculation of junctional conductance (g_j_) from I_j_ recorded at different membrane voltages in Cx43-EGFP yielded similar values within the voltage range illustrated (−40 mV to +40 mV), indicating the absence of voltage dependence (Figure 1C). On the other hand, the g_j_ between HeLa-Cx39 cells at any voltage applied was zero (Figure 1C), revealing the absence of ion flux between contacting HeLa-Cx39 cells.

Cxs that do not form GJCs are not without precedent, since mCx29 (Altevogt et al., 2002) and mCx33 (Chang et al., 1996) have been shown to convey no functional coupling in transfected mammalian cells or in *Xenopus* oocytes. However, positive dye coupling with Alexa488 has been observed in fresh preparations of diaphragm-striated muscle of neonatal mice, composed of myofibers that express only Cx39 (von Maltzahn et al., 2004). Since rhodamine dextran (10 kDa molecular mass) injected into a single myotube failed to spread into neighboring cells (von Maltzahn et al., 2004), the intercellular transfer of Alexa488 is likely to occur through a membrane channel with limited permeability properties. Nonetheless, dye coupling between fully differentiated mouse skeletal muscles has been blocked with suramine, implying the involvement of P2 receptors and perhaps the release and uptake of dye via HCs (Cea et al., 2013). Thus, it is yet to be explored whether a similar mechanism operates in diaphragm striated muscle of neonatal mice. If this is not the case, an alternative possibility could be that one or more factors/conditions for the formation of functional GJCs constituted by endogenously expressed Cx39 might be missing in HeLa cells. Hence, further studies might be necessary to fully rule out or demonstrate whether Cx39 forms GJCs in exogenous expression systems under conditions not studied in the present work (e.g., post-transcriptional modifications not expressed in HeLa cells). Moreover, the possible formation of functional heterotypic channels between homomeric Cx39 channels with other compatible Cx types also remains to be explored.

### Open Cx39 HCs reduce membrane potential and increase total membrane current

**Figure 2:**
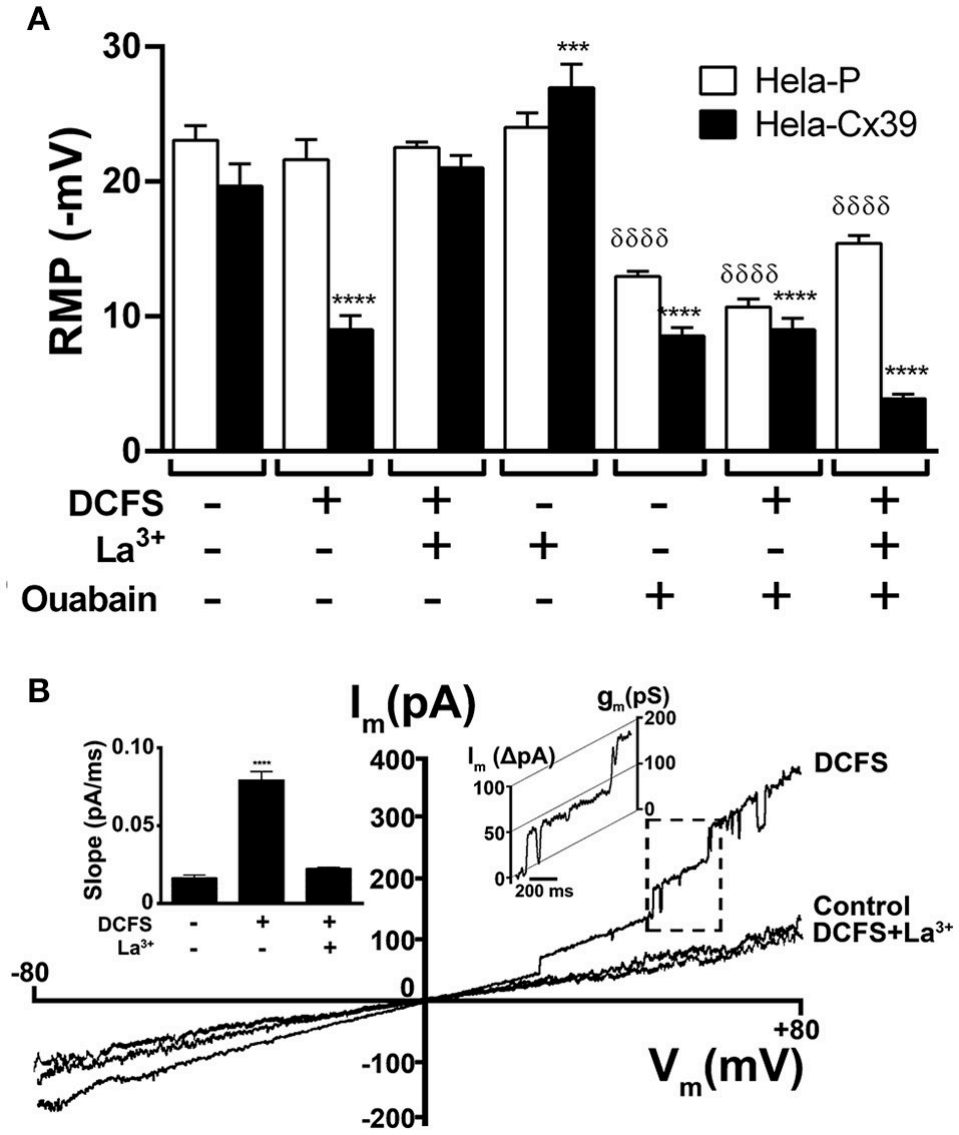
**DCFS reduces the resting membrane potential and increases the macroscopic membrane current of HeLa Cx39 cells. (A)** The RMP of cells was measured using conventional microelectrodes. The graph shows the RMP of HeLa-P and HeLa-Cx39 cells bathed with normal saline solution or DCFS. In parallel experiments cells bathed with normal saline or DCFS were incubated for 30 min with 200 μM La^3+^ or 100 nM ouabain and the RMP was evaluated (^****^p < 0.0001 and ^***^p < 0.0005 compared only between HeLa-Cx39 cells under control conditions v/s all conditions and ^δδδδ^*p* < 0.0001 compared only between HeLa-P cells under control conditions v/s all conditions) *n* = 40 cells. **(B)** Representative membrane I/V curve obtained under application of a voltage ramp from −80 to +80 mV to HeLa-Cx39 cells under control conditions (Control) or DCFS or to DCFS-La^3+^ (200 μM), a HCs blocker. The macroscopic current at V = 0 mV was zero in all conditions. Right inset shows a unitary current event (Rectangle of discontinuous lines) of approximately 75 pS, recorded in the membrane of a HeLa-Cx39 cell. Left inset shows the slope of macroscopic currents under different conditions (*n* = 10, ^****^p < 0.0001).

The low open probability of Cx43 HCs might explain the lack of influence on the RMP in HeLa-Cx43 cells (Contreras et al., 2003). To our knowledge similar studies have not been reported for HCs formed by other Cxs. Thus, as a first approach to study whether Cx39 HCs open under resting conditions, we explored whether they affect RMPs. The RMPs of HeLa Parental cells (HeLa-P cells) in control saline solution or DCFS were similar (23.0 ± 1.1 mV V/S 21.6 ± 1.5 mV, respectively, *n* = 20, *p* > 0.05) (Figure 2A), consistent with the absence of Cx HCs. Accordingly, La^3+^, a HC blocker (Sáez et al., 2005; D'hondt et al., 2009), did not significantly affect the RMPs of HeLa-P cells (−24.0 ± 1.1 mV V/S −23.0 ± 1.1 mV, respectively, *n* = 20 *p* > 0.05) (Figure 2A). Moreover, the RMPs of HeLa-P cells were slightly higher than those of HeLa-Cx39 cells, but the differences were not statistically significant (−23.1 ± 1.1 mV V/S −19.7 ± 1.7 mV, respectively, *n* = 20, *p* > 0.05) (Figure 2A), and the recorded values were similar to those previously measured in HeLa-P and HeLa-Cx43-EGFP cells (Contreras et al., 2003). In control saline solution the application of 200 μM La^3+^ significantly increased the RMPs of HeLa-Cx39 cells, compared with control conditions (−27.0 ± 1.8 mV v/s −19.7 ± 1.7 mV, respectively, *n* = 20 *p* < 0.005), suggesting that inhibition of a few open Cx39 HCs significantly contributed to increasing the electrochemical gradient across the cell membrane. Moreover, the RMPs of HeLa-Cx39 cells bathed in DCFS were significantly lower compared with cells immersed with normal saline solution (−9.0 ± 1.1 mV and −19.7 ± 1.7 mV, respectively, *n* = 20, *p* < 0.0001), indicating that Cx39 HCs are inhibited by a divalent cation likely to be Ca^2+^, as demonstrated for other HCs (DeVries and Schwartz, 1992; Pfahnl and Dahl, 1999; Kondo et al., 2000; Contreras et al., 2003; Gómez-Hernández et al., 2003; Sáez et al., 2005; Lopez et al., 2014; Zonta et al., 2014). Upon exposure to DCFS containing La^3+^ (200 μM), the RMPs of HeLa-Cx39 cells bathed in DCFS were close to those recorded under control conditions (−21.0 ± 1.0 mV v/s −19.7 ± 1.7 mV, respectively, *n* = 20 *p* > 0.05) (Figure 2A), suggesting that the inhibitory effect of La^3+^ predominates. Reductions in RMPs in HeLa-Cx39 cells exposed to DCFS are likely to be mediated by open Cx39 HCs that allow the transmembrane passage of ions (e.g., Na^+^ and K^+^). Accordingly, HCs formed by other Cxs have been shown to be permeable to Na^+^ (Li et al., 2001) and K^+^ (Miro-Casas et al., 2009), and we found that Cx39 HCs are also non-selective channels. Thus, transmembrane Na^+^ and K^+^ gradients drive their diffusional movement, reducing the RMPs to new steady-state values. Although previous works have proposed a gating mechanism activated by Ca^2+^ (Gómez-Hernández et al., 2003; Lopez et al., 2014), recently a Ca^2+^ bound-Cx26 structure revealed that Ca^2+^ is coordinated at the M1/E1 boundary by residues from adjacent subunits, and was propose that Ca^2+^ binding functions as an electrostatic switch that dramatically reduces cation permeability (Bennett et al., 2016). Clearly, more studies are required to explain the mechanism of action of extracellular Ca^2+^ on Cx HCs but independent of this, we found a significant reduction in open probability of Cx39 HCs in Ca^2+^ containing extracellular solution (see below).

In most cells, the Na^+^/K^+^ ATPase pump contributes to the maintenance of RMPs, and its inhibition causes depolarization (Redmann and Walliser, 1981). In agreement with the role of the Na^+^/K^+^ ATPase on the RMP, 30 min treatment in control saline solution or in DCFS with ouabain (0.1 μM), which is a Na^+^/K^+^ ATPase pump inhibitor (Clausen et al., 1983), significantly reduced RMPs to a similar extent in HeLa-Cx39 cells (−8.5 ± 0.6 mV and −9.0 ± 1.1 mV, respectively V/S −19.7 ± 1.7 mV in control saline solution, *n* = 20 *p* < 0.0001) and HeLa-P cells (−10.7 ± 0.6 mV and −13.0 ± 0.4 mV, respectively V/S −23.0 ± 1.1 mV *n* = 20 *p* < 0.0001) (Figure 2A). In addition, Cx39 HC opening in DCFS together with ouabain caused a similar fall in RMPs to that caused by each treatment alone (Figure 2A), suggesting that intrinsic features of the cells prevent a complete drop in RMPs. A possible cause might be the presence of intracellular non-diffusible and non-transportable charged molecules and/or the presence of other compensatory ion transporters. Surprisingly, treatment with La^3+^ plus ouabain caused a further reduction in RMP as compared to treatment ouabain alone, which we cannot explained and requires future studies

To further test that DCFS increases the open probability of Cx39 HCs, we measured the macroscopic and microscopic membrane current of HeLa-Cx39 cells using the whole-cell voltage clamp configuration. The membrane current recorded (Figure 2B) and the current slope calculated from curve current v/s time (Figure 2B, left inset) under control conditions was low and similar to that of HeLa-P cells (not illustrated) (0.016 ± 0.008 pA/mS V/S 0.013 ± 0.005 pA/ms, respectively *p* > 0.05 *n* = 10). However, the membrane current of HeLa-Cx39 cells significantly increased upon exposure to extracellular DCFS, particularly at positive membrane potentials (> +25 mV) and drastically decreased upon 200 μM La^3+^ treatment (0.080 ± 0.006 pA/ms V/S 0.020 ± 0.001 pA/ms, respectively, *n* = 5 *p* < 0.0001). It should be noted that the I/V curve recorded in the presence of extracellular divalent cations (control conditions) was comparable to that recorded in DCFS plus La^3+^ (Figure 2B), suggesting that most, if not all, the increase in membrane current induced by DCFS was due to Cx39 HC opening. The increase in macroscopic currents induced by DCFS was reverted by La^3+^ at basal macroscopic current level in all conditions, at 0 mV, I = 0, indicating that Cx39 HCs have no ionic selectivity like Cx43 HCs (Kondo et al., 2000; Contreras et al., 2003).

Upon application of voltage ramps, the membrane current curves showed unitary events that were frequently observed at positive potentials in HeLa-Cx39 cells exposed to DCFS (Figure 2B). Values of discrete current transitions were point-by-point converted to conductance values, revealing values of unitary events of ~75 pS (Figure 2B, inset in the right panel of the graph). In DCFS, the open probability of unitary current events increased with respect to control conditions, and indicated that the most frequent unitary events was 75 ± 5 pS. The opening and closure transition times of Cx39 HCs were slower than those of Cx43-EGFP HCs in DCFS, but Cx39 HCs had the same open and closure times (Figure 2B, left inset). Similar values of transition times have been observed previously in Cx43-HCs (Contreras et al., 2003) and Cx56-HCs (Ebihara et al., 1999). In HeLa-P cells the application of DCFS did not induce the appearance of discrete unitary current events, as previously reported (Contreras et al., 2003; Retamal et al., 2007; Sánchez et al., 2009).

**Figure 3:**
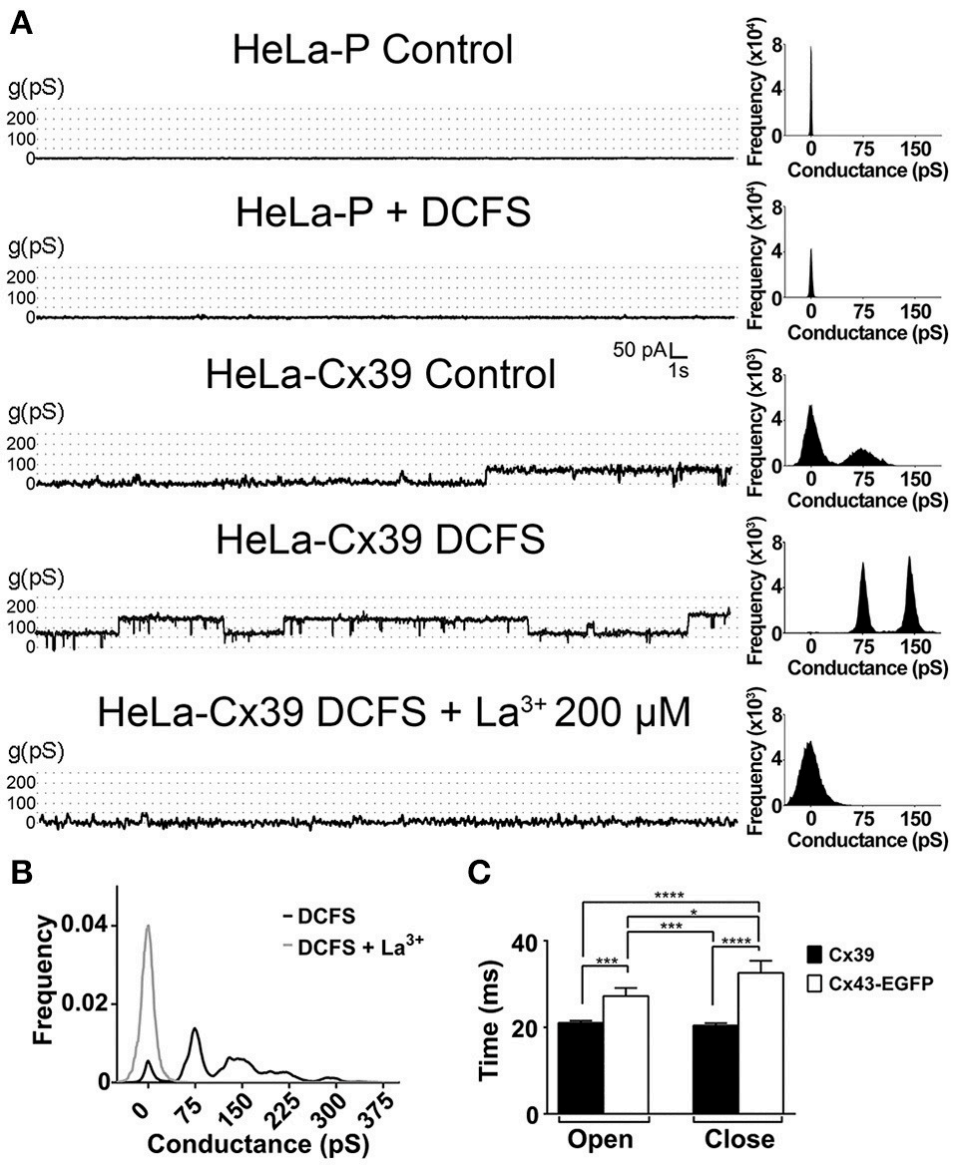
**DCFS increases the open probability of Cx39 HCs. (A)** Unitary current events recorded under voltage clamp at +60 mV in HeLa-P (two top traces) or HeLa-Cx39 cells under the indicated conditions. The frequency of events recorded under each condition is shown in the right of each trace. **(B)** Conductance frequency chart obtained in HeLa-Cx39 cells in DCFS. The most frequent unitary conductance event recorded was 75 ± 5 pS in cells bathed with DCFS (*n* = 40 cells), and was blocked by La^3+^ (*n* = 10). **(C)** Delay times of aperture and closure of Cx39 and Cx43-EGFP HCs. (^*^P < 0.05; ^***^P < 0.0005; ^****^P < 0.0001, *n* = 30).

The unitary current events were also evaluated using rectangular voltage steps. HeLa-P cells held at +60 mV in either control saline solution or DCFS did not elicit unitary current events (Figure 3A). Similar results were obtained in HeLa-P cells transfected with the empty vector (not illustrated). In contrast, in HeLa-Cx39 cells unitary events with a rather low open probability (*P*_o_ = 0.3) were evident after holding the membrane voltage at +60 mV for about 20 s. However, in cells exposed to DCFS, discrete unitary current events showed a higher open probability (*P*_o_ = 0.9), and were completely blocked by 200 μM La^3+^ (Figure 3A). The most frequent discrete event recorded in HeLa-Cx39 cells presented unitary events values of ~75 ± 5 pS (*n* = 40) either in control or DCFS solution (Figures 3A,B). The transition times from the full-closed to full-open states, and vice-versa, of Cx39 HCs were not significantly different (21.0 ± 0.5 ms v/s 20.4 ± 0.5 ms, *P* > 0.05, *n* = 30) (Figure 3C), but both values were lower than those of Cx43-EGFP HCs (full-closed to full-open: 27.2 ± 1.9 ms v/s 32.6 ± 2.8 ms, *P* < 0.05, *n* = 30 and full-open to full-closed: 20.4 ± 0.5 ms v/s 32.6 ± 2.8 ms, *P* < 0.0001, *n* = 30) (Figure 3C).

### Cx39 HCs enable the passage of a selective group of molecules with different physicochemical properties

**Figure 4:**
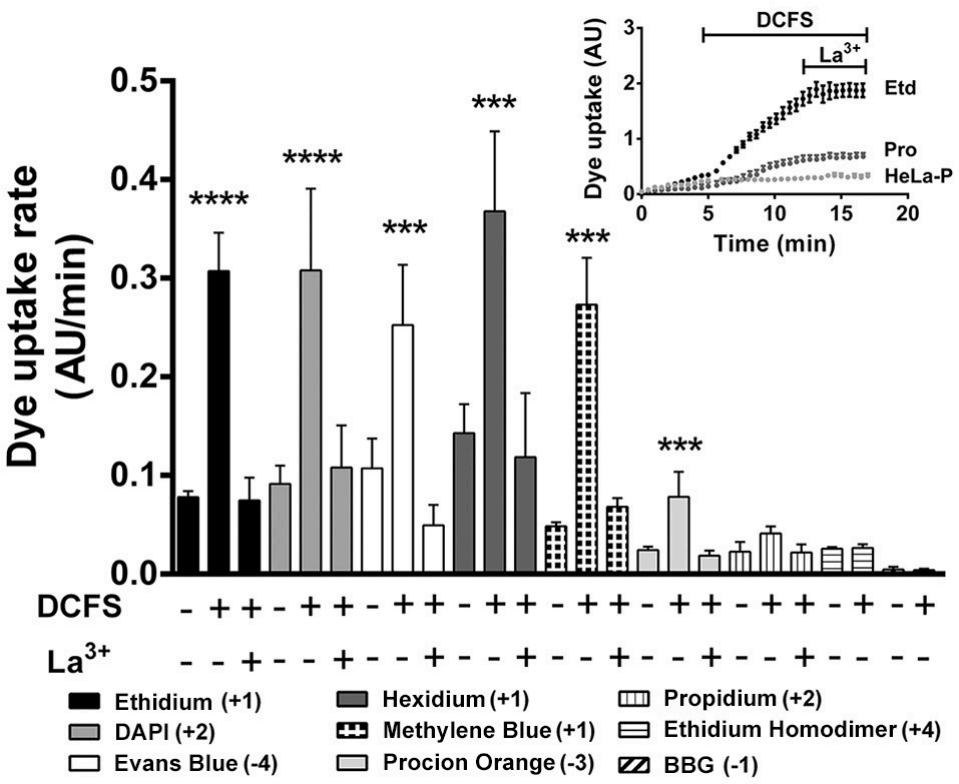
**The dye uptake of HeLa-Cx39 cells is increased by DCFS**. Uptake rate of the different molecules with different molecular mass and net charge (Bottom list) of HeLa-Cx39 cells under each condition indicated (*n* = 6 for each dye). **Inset** shows representative real time ethidium (Etd^+^) and propidium (Pro) uptake, HeLa-Cx39 cells respectively, and Etd^+^ in HeLa-P. After 5 min recording under control conditions, the extracellular solution was quickly changed by DCFS and dye uptake was recorded for 8 min followed by additional 5 min recording in the presence of 200 μM La^3+^. (^****^P < 0.0001 and ^***^p < 0.0005 compared to basal value in each case; *n* = 4, with 30 cells recorded in each case). AU, arbitrary Units.

Different dyes (e.g., DAPI^+2^, Etd^+^ and propidium) have been used before to observe changes in membrane permeability due to the presence of Cx HCs (Orellana et al., 2011; Hansen et al., 2014). However, the selectivity of the Cx pores is not easily visualized with a short list of permeability probes. To identify permeability features of Cx39 HCs, we performed real-time dye uptake experiments using molecules with different molecular masses and net charges that fluoresce upon binding to intracellular nucleic acids and proteins (Table 1 and Figure 4).

HeLa-P cells did not show changes in ethidium uptake upon exposure to DCFS (Figure 4, inset). Similarly HeLa-Cx39 cells in the presence of extracellular divalent cations showed very low uptake of ethidium (+1) and propidium (+2). After exposing HeLaCx39 cells to DCFS, only ethidium uptake increased drastically and was completely blocked by 200 μM La^3+^ (Figure 4, inset). In fact, the uptake rate of all dyes used in HeLa-Cx39 cells was very low under control conditions, but the uptake rate of several dyes increased significantly upon exposure to DCFS; Etd^+^, DAPI (+2), Evans blue (−4), hexidium iodide (+1), methylene blue (+1) and procion orange MX2R (−3), and was canceled out by La^3+^ (Figure 4). In contrast, the uptake rate of propidium (+) showed a tendency to increase in DCFS that was reduced by La^3+^ (not statistically significant), whereas the uptake rate of ethidium homodimer (+4) and BBG (−1) was not significantly affected either by DCFS or La^3+^ (Figure 4). In agreement with the lack of HCs, the dye uptake rate of HeLa-P cells was almost zero in either control saline solution or after exposure to DCFS alone or with La^3+^ (not shown).

The permeability of Cx39 HCs to molecules varied between large molecules with high net charge, like TOTO (MW: 1,300, +4), YOYO (MW: 1,200, +4) or Evans blue (MW: 960, −4), to smaller molecules with lower net charges, which are not able to pass through HCs-Cx39, such as ACMA dye (MW: 258, +1) or with high net charge, such as ethidium homodimer (MW: 857, +4). Because permeable dyes differ in terms of their molecular nature (Table 1), explaining why a dye can pass through Cx39 HCs depends on considering the nature of the Cx39 HC and the molecular features of the dyes. Since the above findings suggest a lack of correlation between size and charge, we decided to use a machine-learning approach to analyze the molecular features of permeant molecules in order to further understand the permeability properties of Cx39 HCs (see below).

### Electronegativity, ionization potential, flexibility, valence, size, polarizability and geometry are key features of small molecules to define their passage through mCx39 HCs

Determining permeability features with various dyes could provide useful information about the physical and chemical nature of the pores and their affinity to certain molecules, according to their net charge, size or shape. However, these selection criteria appear to be insufficient to identify the basic features of molecules that cross the cell membrane through Cx39 HCs (see above). To overcome this limitation, we used a machine-learning approach to explore the physicochemical properties of the different permeability probes tested in the present work. We identified 11 out of 1,000 molecular descriptors (Table 2) that correctly classified the dyes in terms of their ability to permeate mCx39 HCs (Table 1). Among the selected physicochemical properties, the most frequent belong to six different categories: (1) electronegativity, (2) ionization potential, (3) polarizability, (4) size and geometry, (5) flexibility and (6) valence. While the values of some molecular descriptor were higher (TDB6e, ATSC5s, GATS5s, TDB8i, TDB10i, TDB6v, and TDB5u) those of others were smaller (RotBtFrac and VP-6) for permeant molecules as compared to non-permeant molecules (Table 1). All other descriptors did not follow this pattern and, thus, were not included in Table 2 (Table S1). An extended explanation for each descriptor can be reviewed in the Supplementary Materials and Methods section. Since some values of several descriptors are common features to all permeant molecules, while others are characteristic of non-permeant molecules, the value of one descriptor alone is not enough to establish whether a particular molecule is permeant or not. Therefore, the permeability of dyes could be understood as an emergent property obtained from a non-linear combination of several physicochemical properties: electronegativity, ionization potential, flexibility, valence, size, polarizability and geometry. Despite non-linearity, still some relationships between these properties could be obtained. As electronegativity is proportional to valence, it is expected that descriptors TDB6e, ATSC5s, GATS5s and VP-6 would show similar trends (i.e., electronegative patches are spread and separated, and low valence atoms are close to each other). Procion orange MX2R and Evans blue have many ${\text{SO}}_{3}^{-}$ groups distributed throughout their structure, and permeate through mCx39 HCs. BBG also has a couple of ${\text{SO}}_{3}^{-}$ groups, but as discussed below, size-related descriptors hint as to why it does not permeate. Ionization potentials (8i/10i) and polarizability (TDB6p) also show similar trends, mainly due to the fact that most tested dyes have resonance structures that allow electron delocalization (i.e., polarizability spread throughout the molecule). In the case of propidium, although it has similar resonance structures like ethidium and hexidium, it possesses two positive charges close to each other. This is the contrary to what happens for DAPI^+2^, since its two positive charges are far from each other. Interestingly, molecular flexibility tends to be inversely related to dye diffusion through Cx39 HCs: dyes with lowest uptake rates (Propidium, ethidium homodimer and BBG) have the highest values for the RotBtFrac descriptors (Table 2). This notion is reinforced because, on a visual inspection, BBG and the ethidium homodimer present bulkier structures than the rest of the molecules (as suggested by descriptors TDB6r, TDB6v, and TDB5u), although they have similar molecular weights and van der Waals volumes to Evans blue (data not shown). Moreover, DAPI^+2^, Evans blue and methylene blue have an overall linear structure, while Etd^+^ and hexidium iodide are relatively small, which might favor their diffusion through Cx39 HCs. In the case of procion orange MX2R, although being a negatively charged molecule such as Evans blue, it possesses two chloride atoms that may make its diffusion difficult. Pore radius may also be a factor impeding these bulky molecules from diffusing freely through mCx39 HCs. In contrast to other Cx HCs, mCx39 HC exhibits a constriction at the PH region that is absent in hCx26 HC and mCx43 HC. Hence, it is evident that bulkier structures, such as BBG and the ethidium homodimer, will have more difficulty crossing the barrier formed by the PH region of mCx39. As for propidium, although its structure is similar to ethidium and hexidium, it possesses a quaternary ammonium group, which can rotate freely, changing the radius of gyration of the molecule and possibly hindering its diffusion through Cx39 HCs.

**Table 2:** **Raw data of the selected molecular descriptors for each tested molecule**.

**Category B3:L14**	**Descriptor**	**Ethidium bromide**	**DAPI^+2^**	**Evans blue**	**Hexidium iodide**	**Methylene blue**	**Procion orange MX2R**	**Propidium iodide**	**Ethidium homodimer-2**	**BBG**	
Electronegativity	TDB6e	41.75	45.31	53.62	42.18	45.98	51.98	41.28	41.16	40.90	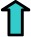
	ATSC5s	−0.13	1.50	−311.14	2.62	1.91	−37.82	9.71	15.42	6.61	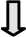
	GATS5s	0.67	0.77	1.25	0.66	0.77	1.07	0.57	0.56	0.54	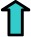
Ionization potential	TDB8i	1,254.65	1,362.00	1,201.18	1,188.58	1,312.73	1,188.57	1,171.03	1,179.16	1,103.96	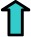
	TDB10i	1,816.65	1,733.37	1,460.62	1,515.93	1,773.06	1,451.99	1,402.39	1,418.41	1,296.55	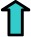
Polarizability	TDB6p	8.32	8.26	8.61	8.51	9.33	10.12	7.71	7.96	7.87	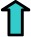
Size and geometry	TDB6r	1.82	1.87	2.29	1.85	2.00	2.49	1.71	1.76	1.71	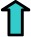
	TDB6v	1,080.21	1,100.05	1,333.31	1,114.43	1,121.27	1,460.71	1,005.28	1,039.38	1,018.14	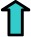
	TDB5u	4.91	4.90	4.99	4.94	4.87	5.04	4.68	4.76	4.80	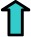
Topological flexibility	RotBtFrac	0.19	0.22	0.30	0.29	0.23	0.27	0.35	0.32	0.34	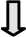
Valence	VP-6	2.15	1.04	2.57	2.72	1.82	1.95	3.15	6.92	3.36	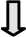

*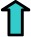 All values of permeable molecules are higher than values of impermeant ones*.

*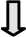 All values of permeable molecules are lower than values of impermeant ones*.

### Open Cx39 HCs do not enable Ca^2+^ influx

**Figure 5:**
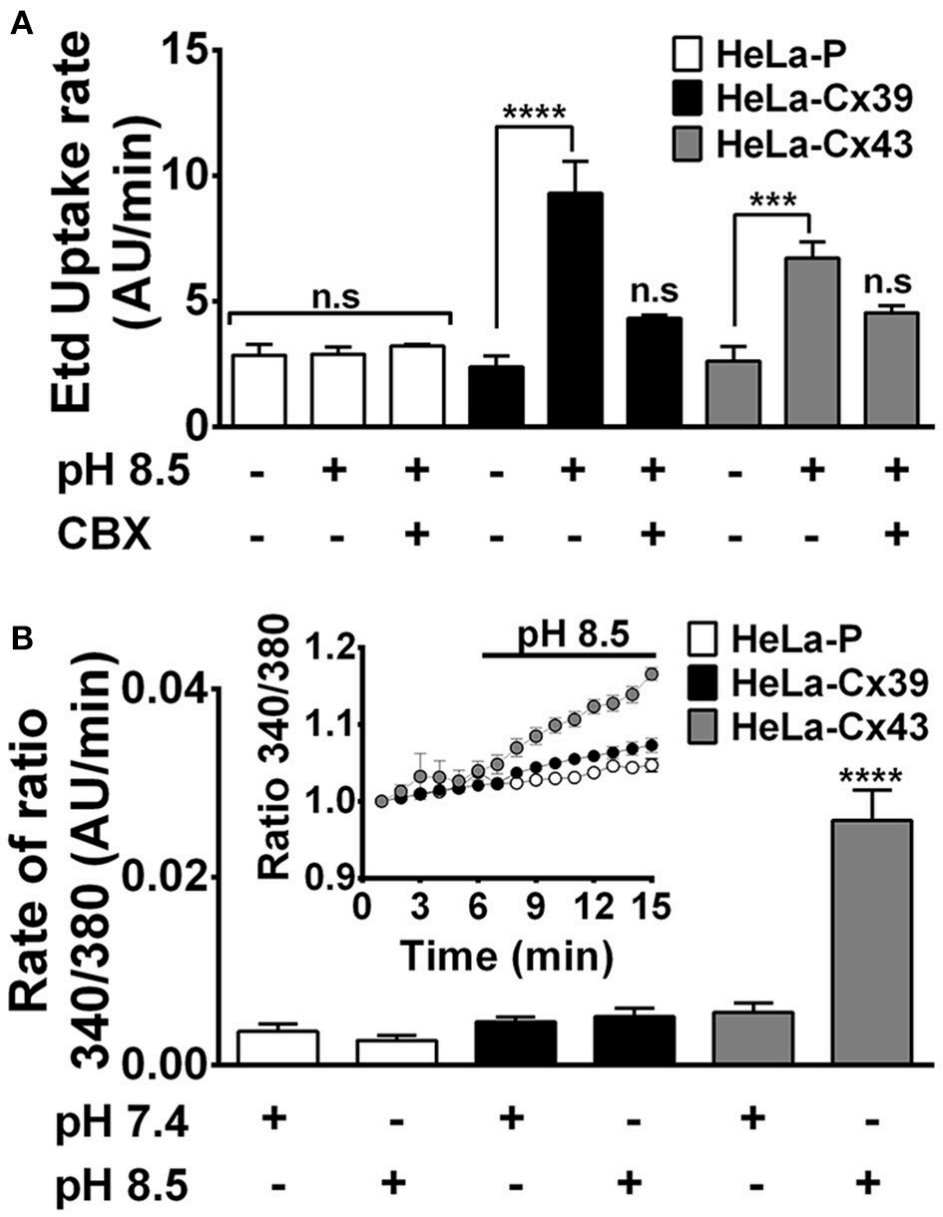
**Alkaline pH opens Cx39 HCs, which are permeable to ethidium but not to Ca^2+^**. HeLa-P, HeLa-Cx39, or HeLa-Cx43 cells were exposed to extracellular saline solution with pH 7.4 or pH 8.5. **(A,B)** Etd^+^ uptake rate and Ca^2+^ signal, respectively, were evaluated over time. **(B) Inset**. Real time of Ca^2+^ signal increase in HeLa-P, HeLa-Cx39 and HeLa-Cx43 cells, bathed with alkaline solution after register in control conditions. ^****^p < 0.0001 and ^***^p < 0.0005 respectively, *n* = 3 experiments and 20 cells were recorded in each experiment. AU, Arbitrary Units.

To evaluate Ca^2+^ uptake, it is necessary to use a stimulus that opens Cx HCs in the presence of extracellular Ca^2+^, as it occurs upon exposure to alkaline pH (Schalper et al., 2010). Thus, we exposed cells to an alkaline saline solution (pH 8.5), and the Etd^+^ uptake and Ca^2+^ signals were simultaneously evaluated. As previously demonstrated, HeLa-Cx43 cells showed an increase in Etd^+^ uptake as well as in Ca^2+^ signal (Figures 5A,B). The same treatment induced Etd^+^ uptake in HeLa-Cx39 cells (9.3 ± 1.3 AU/min v/s 2.4 ± 0.4 AU/min, respectively *p* < 0.0001 *n* = 4) (Figure 5A), but Ca^2+^ signal was not significantly modified (Figure 5B, inset). On the other hand, HeLa-P cells exposed to alkaline pH did not show changes in Etd^+^ uptake and Ca^2+^ signal (Figures 5A,B).

**Figure 6:**
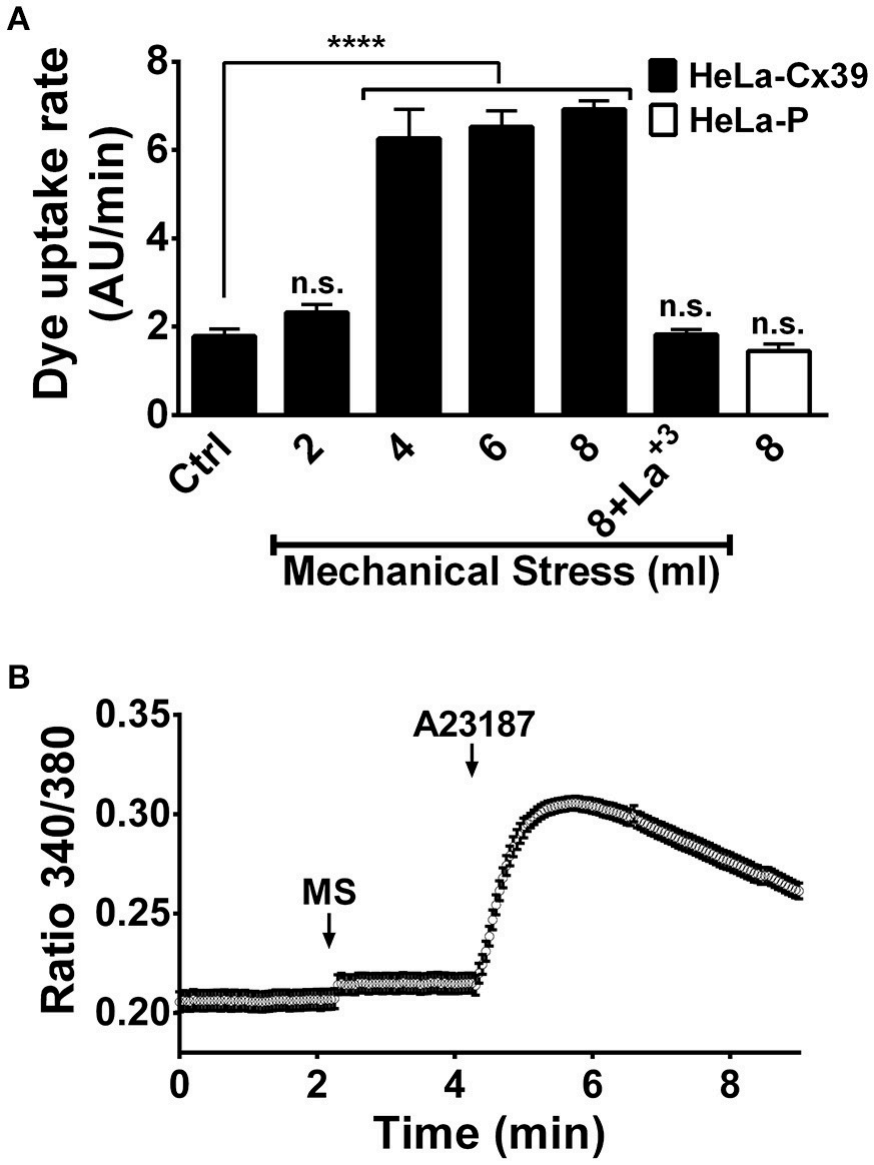
**Cx39 HCs of cells exposed to mechanical stress are permeable to ethidium but not to Ca^2+^. (A)** Dye (Etd^+^) uptake rate of HeLa-P and HeLa-Cx39 cells mechanical stimulated with different volumes of saline solution from 15 cm high under control conditions. HeLa-Cx39 cells were also mechanically stimulated with 8 ml in the presence of 200 μM La^3+^ (8+La^3+^). HeLa-P cells were also stimulated with 8 ml of saline solution. (^****^p < 0.0001, *n* = 5 with 30 cells recorder in each case). **(B)** Intracellular Ca^2+^ signal of HeLa-Cx39 cells under control conditions (first 2.5 min), after mechanical stimulation (8 ml from 15 cm high) and after the addition of 2.5 μM of 4-Bromo calcium ionophore A23187 to increase calcium in the cells. MS, mechanical stress.

To further study the possible permeability of Cx39 HCs to Ca^2+^, we induced HC opening by applying mechanical stress, a stimulus that induces opening of HCs constituted of other Cxs and in other cell types (Stout et al., 2002; Batra et al., 2012). We found a direct relationship between the intensity of mechanical stress and the rate of Etd^+^ uptake in HeLa-Cx39 cells (Figure 6A). While the application of 2 serial stimuli of 1 ml from 15 cm high did not significantly increase dye uptake rate, the application of 4 stimuli caused similar increases in dye uptake rate as those induced by 6 or 8 stimuli (Figure 6A). These increases in dye uptake were completely suppressed by La^3+^ applied during the time-lapse measurement (Figure 6A), indicating that the dye uptake occurred through Cx39 HCs. It should be noted that applying 8 stimuli to HeLa-P cells did not increase dye uptake (Figure 6A), which is consistent with the absence of Cx39 HCs. Then, we evaluated possible Ca^2+^ influx in HeLa-Cx39 cells, and found no increase in intracellular Ca^2+^ signal after mechanical stimulation (Figure 6B). However, the application of the 2.5 μM calcium ionophore A23187 rapidly increased the Ca^2+^ signal that afterwards decreased progressively (Figure 6B), possibly due to the increase in open probability of Cx39 HCs by the increase in intracellular Ca^2+^ induced by the ionophore as observed for other Cx HCs (De Vuyst et al., 2006, 2009), which could favor the leakage of Fura-2 to the extracellular solution. Interestingly Cx39 HCs are the first HC shown to be impermeable to Ca^2+^, suggesting that is opening might less harmful for cells under pathological conditions such in denervation skeletal muscle (Cea et al., 2013) and in Duchenne muscular dystrophy (Cea et al., 2016)

### Dye uptake of Hela-Cx39 cells is sensitive to some, but not all, HC blockers

**Figure 7:**
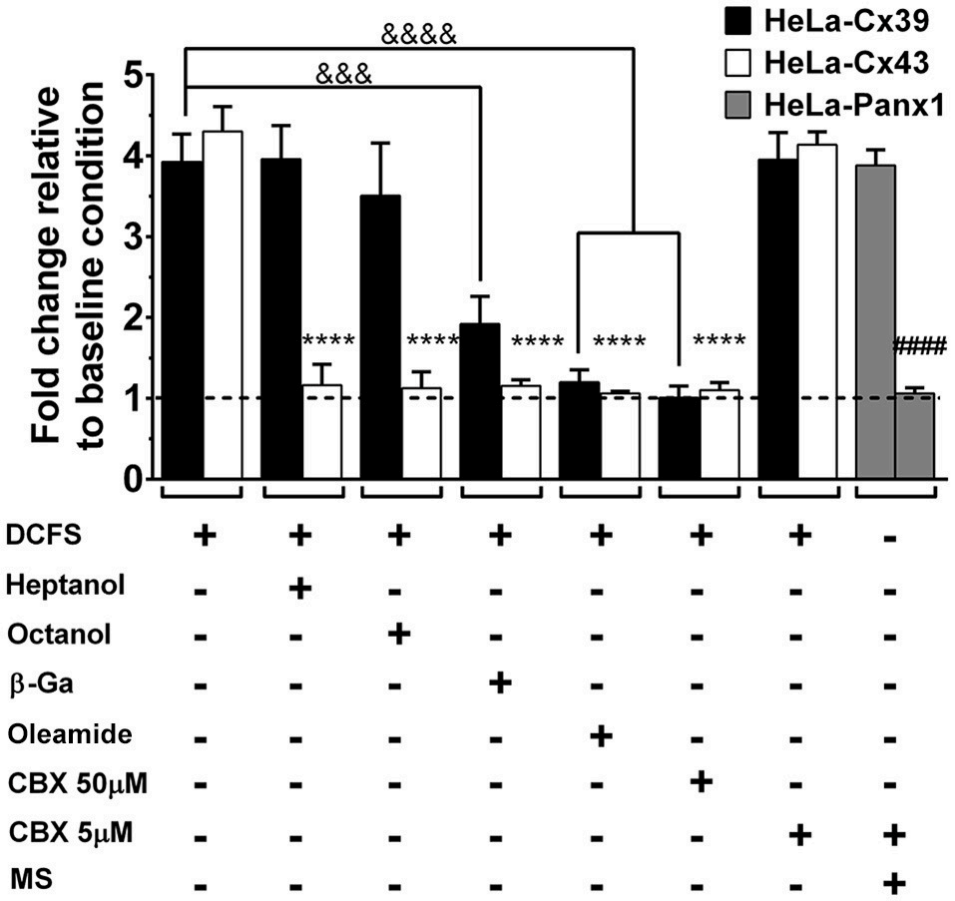
**Cx39 HCs are inhibited by some conventional Cx HCs blockers**. The graph shows normalized data with respect to the basal condition of Etd^+^ uptake rate induced by DCFS in HeLa-Cx43 EGFP and HeLa Cx39 cells. Heptanol (350 μM), octanol (310 μM), 18β-glycyrrhetinic acid (50 μM, β-GA), oleamide (100 μM) or carbenoxolone (5 to 50 μM, CBX) was applied together with DCFS (*n* = 10, ^&&&&^*p* < 0.0001, ^&&&^*p* < 0.0005 and ^****^p < 0.0001 compared only between HeLa-Cx39 cells and HeLa-Cx43 EGFP respectively, under increased dye uptake induced by DCFS v/s all dye uptake blockers; ^####^*p* < 0.0001 compared only between HeLa-Panx1 cells).

Several GJ and HC blockers have been described and their properties have been reviewed (Hervé and Sarrouilhe, 2005; Bodendiek and Raman, 2010; Verselis and Srinivas, 2013). Although Cx39 HCs (Figures 2B, 4 inset) and Cx43 HCs (Contreras et al., 2003) are rapidly blocked by La^3+^ in cells exposed to DCFS, the application of 310 μM octanol, 350 μM heptanol, 50 μM β-glycyrrhetinic acid, 100 μM oleamide and 50 μM carbenoxolone did not block after inducing HC opening by DCFS. This suggests that these agents do not block open Cx39 or Cx43 HCs. However, co-application of 50 μM β-glycyrrhetinic acid, 100 μM oleamide or 50 μM carbenoxolone with DCFS to HeLa-Cx39 and Cx43 HeLa-EGFP cells significantly precluded the dye uptake increase induced by DCFS in both HeLa-Cx43 and -Cx39 cells, suggesting that they block Cx39 HCs in the closed state. In contrast, heptanol and octanol did not prevent DCFS-induced dye uptake in HeLa-Cx39 cells (Figure 7). Nevertheless, these two alcohols effectively prevented the DCFS-induced opening of Cx43 HCs (Figure 7). The action of long-chain alcohols like heptanol has been explained by their effects on membrane fluidity (Bastiaanse et al., 1993), while a specific and high-affinity interaction with Cx50 HCs has been reported for octanol (Eskandari et al., 2002) the lack of such a high-affinity domain in Cx39 HC might explain the insensitivity of this HC to heptanol and octanol.

The structurally related classical Cx HC blockers, β-glycyrrhetinic acid and carbenoxolone, partially and completely blocked the activity of Cx39 HCs (Figure 7). These derivatives seem to affect many different GJCs without being specific to any Cx subtype, and the proposed mechanism of action would involve the insertion of glycyrrhetinic derivatives into the plasma membrane, hence binding to the GJC subunits at hydrophobic sites and inducing a conformational change that leads to channel closure (Davidson et al., 1986).

Since carbenoxolone (50 μM) is known to block Cx and Panx1 HCs (Bruzzone et al., 2005), which are rather insensitive to heptanol (Pelegrin and Surprenant, 2006), the possibility that Cx39 HCs present pharmacological sensitivity similar to Panx1 HCs was also studied. To this end, we tested whether a low carbenoxolone concentration (5 μM CBX), which is a classical Panx1 HC blocker (Bruzzone et al., 2005; Ma et al., 2009), affects the functional state of Cx39 HCs. Opening of Panx1 HCs was induced with mechanical stress in HeLa-Panx1 cells used as controls (Bao et al., 2004; Locovei et al., 2006; Taylor et al., 2015), and 5 μM CBX drastically reduced the dye uptake. In contrast, 5 μM CBX did not affect the mechanical stress-induced dye uptake in HeLa-Cx39 cells (Figure 7).

### Molecular modeling of Cx39 and Cx43 HCs

**Figure 8:**
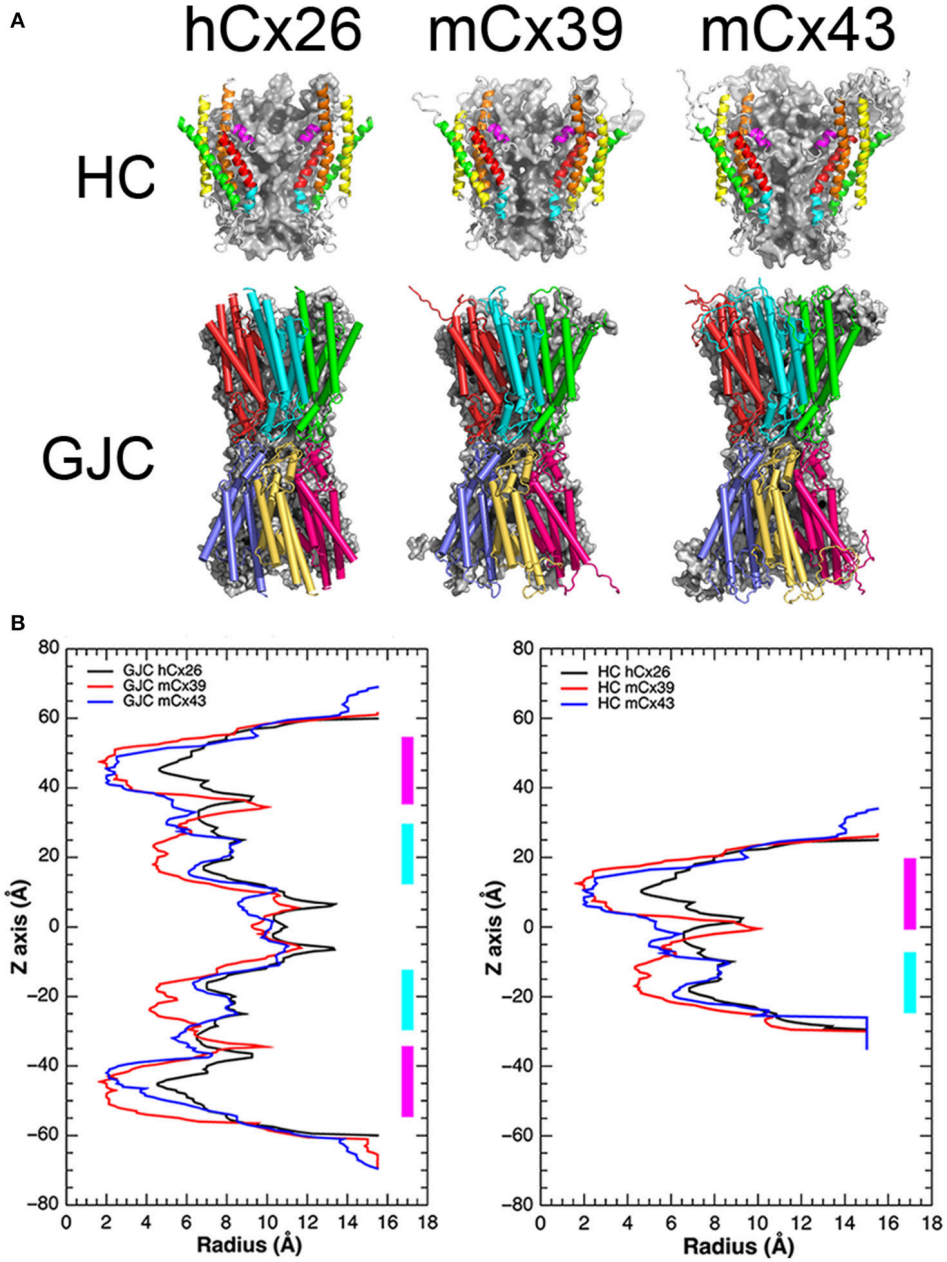
**mCx39 resemble a Cx based channel but exhibit structural differences in PH region compared with mCx43 an hCx26. (A)** Top row, structural representations of the hCx26 crystal structure of the HC used as a template, and the molecular models of mCx39 and mCx43. Each HC shows two monomers that are depicted by ribbons and color coded to represent different regions of the protein (NTH = magenta, TM1 = red, PH = cyan, TM2 = orange, TM3 = yellow, and TM4 = green). Two other monomers are depicted in a gray solid surface, while the other two at the front were omitted for clarity. **(A)** Bottom row, structural representations of the hCx26 GJC used as template, and the molecular models of mCx39 and mCx43. Each HC shows three monomers that are depicted by cartoons (cylinders = alpha helices, arrows = beta sheets, tubes = coils and loops) and color coded to differentiate from each other. **(B)** Pore radius along the z-axis of the Cx channels. Left, pore radius along the z-axis calculated for the hCx26 crystal structure (black) of the GJCs and the mCx39 (red) and Cx43 (blue) molecular model. Right, pore radius along the z-axis calculated for the hCx26 crystal structure (black) of the HC and the mCx39 (red) and Cx43 (blue) molecular models. The NTH region is depicted in magenta while the PH regions appears in cyan.

To gain insight into the structural and/or functional differences between mCx39 and mCx43, which may help us explain why the former are not able to produce functional GJCs in Hela cells, we developed a set of molecular models for both HCs and GJCs based on the hCx26 crystal structure as a template (Figure 8A). As expected and independent of individual sequence variations, all models resemble the general structure of the hCx26 channel. However, some differences in the pore structure along the channel can be found (Figure 8A, top row). To determine if these structural differences may influence channel function, the pore radius for all HCs and GJCs was computed (see Materials and Methods). The pore radius of hCx26 in both GJCs (Figure 8B left) and HCs (Figure 8B right) shows the narrowest constriction at the N-terminal helix (NTH) region, which reaches 4.5 Å, while the widest part is found at the extracellular side of the Para-Helix (PH) region, with a pore radius near 14 Å. The pore radius calculated for mCx39- and mCx43-based channels follows a similar tendency, showing the narrowest part of the pore at the NTH region, reaching a radius less than 2 Å. Both channels exhibit the widest part of their pore at the extracellular side of the PH region, similar to hCx26-based channels, reaching a radius near to 12 Å. Of note, the pore radius of mCx39 in GJCs (Figure 8B left) and HCs (Figure 8B right) exhibits a notorious constriction, which is absent in the other structures: a pore radius near 4 Å at the intracellular side of the PH region.

According to the authors of the crystal structure of hCx26, this channel was obtained in its open state (Maeda et al., 2009). However, molecular dynamics (MD) simulations performed by Kwon et al. (2011) using the crystal structure of the hCx26 HC concluded that this structure is unlikely to represent an open channel. Moreover, later on, Zonta et al. (2013) reached the same conclusion: the hCx26 crystal structure should be in its closed conformation. Despite this controversy, available experimental evidence suggest that the NTH region is related to voltage-dependent fast-gating of the channel (Oh and Bargiello, 2015). Therefore, we hypothesized that any restriction present in this region could be affected by the relocalization of the NTH in the presence of a transmembrane potential difference. This notion is supported by previous work that suggests that the voltage sensor can be an integral part of the NTH (Ek-Vitorin and Burt, 2013; Araya-Secchi et al., 2014).

On the other hand, mCx39 exhibits a singular constriction at the intracellular side of the PH region that is absent in the mCx43 and hCx26 template structure. Similar to NTH, this region has also been related to voltage-dependent gating of Cx-based channels, namely the slow gating (Oh and Bargiello, 2015). As mentioned before, HeLa cells expressing Cx39 do not form functional GJCs. Therefore, we hypothesize that the constriction present in mCx39 at the PH region could respond to a transmembrane voltage difference. If this is the case, apart from the apparent steric restriction, the PH region should also present other features that may explain lack of GJC functionality.

Interestingly, the recent release of a hCx26 crystal structure obtained in the presence of extracellular Ca^2+^, suggest that despite both regions the NTH and the PH could respond to voltage differences across the cellular membrane, the Ca^2+^ induced channel blockage could be the result of an electrostatic blockage instead of a set of conformational changes (Bennett et al., 2016).

**Figure 9:**
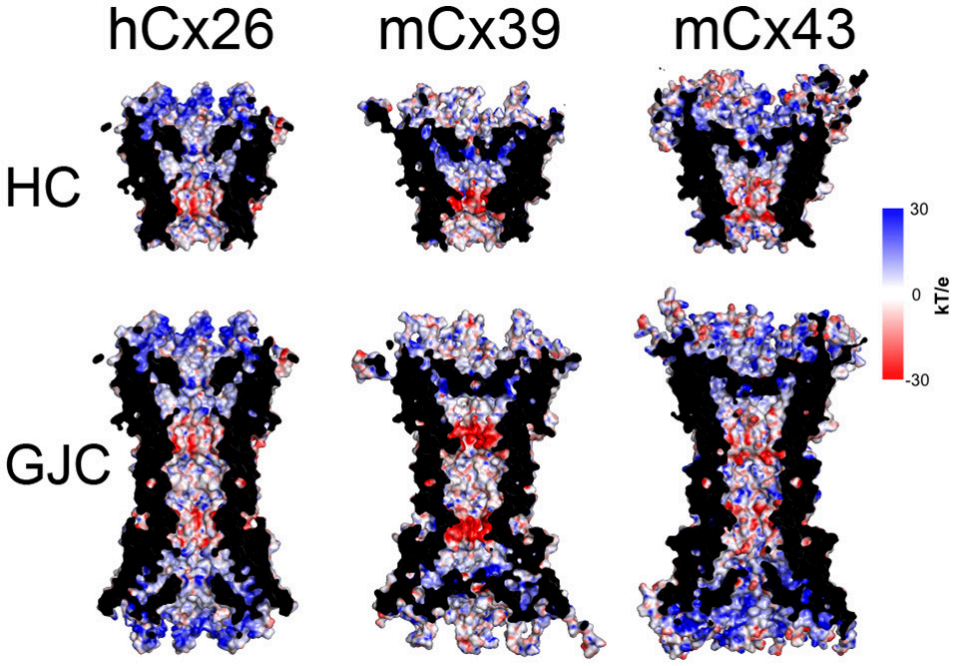
**Molecular surface along the pore of Cx-based channels colored by electrostatic potential**. **Top row**, the electrostatic potential mapped onto the molecular surface of the hCx26 crystal structure of the HC used as a template, and the mCx39 and mCx43 molecular models. **Bottom row**, the electrostatic potential mapped onto the molecular surface of the hCx26 crystal structure of the GJC used as a template, and the mCx39 and mCx43 molecular models. All structural representations depict 4 monomers per HC while the other two at the front were omitted for clarity. Color coding corresponds to the value of the electrostatic potential according to the spectrum shown at the right (in kT/e units).

To further explore the implications of these structural differences between mCx39 and mCx43, considering their sequence variation, we decided to compute the electrostatic potential of our molecular models. The electrostatic potential of both hCx26 and mCx43, exhibits a general trend. The intracellular portion shows an electropositive confluence, whereas the extracellular side tends to be electronegative, showing a diffuse pattern in which negative charges are mixed primarily with neutral regions and, to a lesser extent, with positive regions (Figure 9). Interestingly, mCx39 escapes this trend by exhibiting a more neutral intracellular side, showing a diffuse pattern of positive charges, and a markedly electronegative confluence at the extracellular side flanked by two neutral regions located at both sides of this negative charge accumulation (Figure 9). Moreover, this electronegative patch co-localizes with the unique constriction exhibited by mCx39 at the intracellular region of the PH (Figure 9). Considering the importance of the localization of charges for the ionic selectivity of Cx-based channels (Ek-Vitorin and Burt, 2013; Escalona et al., 2016) it is expected that any alteration to this pattern could hinder ion currents or voltage sensing, hence affecting channel function. A possible functional consequence of the homotypic docking could be cell–cell addition, which is an important Cx function frequently overlooked

## Conclusions

Results reported here demonstrate that Cx39 forms functional HCs with distinct unitary conductance (75 ± 5 pS) and inhibited by extracellular Ca^2+^, however it does not form functional GJCs. Cx39 HCs are preferentially permeable to molecules characterized by six categories of descriptors, namely (1) electronegativity, (2) ionization potential, (3) polarizability, (4) size and geometry, (5) topological flexibility and (6) valence. Unlike other Cx HCs, Cx39 HCs are not permeable to Ca^2+^ and show different in sensitivity to classical HC and GJC blockers.

## Author contributions

BAC performed permeability experiments, FS performed electrophysiological experiments, CU and LAC performed permeability experiments, AHV designed and performed electrophysiological experiments. SEGM, AJMM analyzed bioinformatics data of dyes and wrote the paper, CPB, YE performed *in silico* model and wrote the paper, TPA designed bioinformatics experiments and wrote the paper, OS and CFL wrote the paper, and AAV designed and performed electrophysiological and permeability experiments and wrote the paper and JCS designed research and wrote the paper.

### Conflict of interest statement

The authors declare that the research was conducted in the absence of any commercial or financial relationships that could be construed as a potential conflict of interest.
