# Supplementary material for: On Biophysical Properties and Sensitivity to Gap Junction Blockers of Connexin 39 Hemichannels Expressed in HeLa Cells
Source: Front Physiol. 2017 Feb 9;8:38. doi: 10.3389/fphys.2017.00038 (PMC5298994; doi:10.3389/fphys.2017.00038)
Supplement: Supplementary file 2 [file DataSheet1.PDF]

Supplementary Materials and Methods

Descriptors ATSC5s, AATSC1v, MATS1v, GATS5s belong to a greater group known as Autocorrelation of a Topological Structure (ATS), and generally describe how a certain property is distributed along the topological structure of the molecule [1]. In this case, ATSC5s and GATS5s refer to the intrinsic state, which is related to the valence state electronegativity of the atom, while AATSC1v and MATS1v refer to the van der Waals volumes of the atoms [1].

Descriptor VP-6, which stands for Valence Path of order 6, corresponds to the path-subtype Chi index. The Chi indexes characterize structural attributes of molecules by taking into account the total number of valence electrons of each atom and the number of covalent bonds between them [2].

Descriptor RotBtFrac denotes the fraction of rotatable bonds (including terminal bonds) and is related to the internal flexibility of the molecule, and thus, its shape.

TDB descriptors correspond to 3D autocorrelations based on topological distances within the molecule. Although similar to ATS descriptors, TDB depend on the interatomic distances instead of the topological ones, but are still dependent on the same set of atomic properties. In this case, the considered properties were van der Waals volume (v), electronegativity (e), polarizability (p), covalent radius (r) and first ionization potential (i, which describes the energy required to remove the outer most electron to form a cation), as well as a more general term describing only its geometry (u, which stands for *unweighted*) [1].

REFERENCES

1. Todeschini, R., et al., *Molecular descriptors for chemoinformatics, volume I: alphabetical listing/volume II: appendices, references; methods and principles in medicinal chemistry (volume 41)*. 2009, Wiley-VCH, Weinheim.
2. Hall, L.H. and L.B. Kier, *The Molecular Connectivity Chi Indexes and Kappa Shape Indexes in Structure-Property Modeling.* Reviews in Computational Chemistry, Volume 2, 2007: p. 367-422.
